# Reversal of Cognitive Impairment in gp120 Transgenic Mice by the Removal of the p75 Neurotrophin Receptor

**DOI:** 10.3389/fncel.2019.00398

**Published:** 2019-08-30

**Authors:** Andrew Speidell, Gino Paolo Asuni, Valeria Avdoshina, Serena Scognamiglio, Patrick Forcelli, Italo Mocchetti

**Affiliations:** ^1^Laboratory of Preclinical Neurobiology, Department of Neuroscience, Georgetown University Medical Center, Washington, DC, United States; ^2^Department of Pharmacology and Physiology, Georgetown University Medical Center, Washington, DC, United States

**Keywords:** HIV, Morris water maze, NMDA receptors, p75NTR, passive avoidance, proBDNF, PSD95

## Abstract

Activation of the p75 neurotrophin receptor (p75NTR), by the proneurotrophin brain-derived neurotrophic factor (proBDNF), triggers loss of synapses and promotes neuronal death. These pathological features are also caused by the human immunodeficiency virus-1 (HIV) envelope protein gp120, which increases the levels of proBDNF. To establish whether p75NTR plays a role in gp120-mediated neurite pruning, we exposed primary cultures of cortical neurons from *p75NTR*^–/–^ mice to gp120. We found that the lack of *p75NTR* expression significantly reduced gp120-mediated neuronal cell death. To determine whether knocking down *p75NTR* is neuroprotective *in vivo*, we intercrossed gp120 transgenic (tg) mice with *p75NTR* heterozygous mice to obtain gp120tg mice lacking one or two *p75NTR* alleles. The removal of *p75NTR* alleles inhibited gp120-mediated decrease of excitatory synapses in the hippocampus, as measured by the levels of PSD95 and subunits of the *N*-methyl-D-Aspartate receptor in synaptosomes. Moreover, the deletion of only one copy of the *p75NTR* gene was sufficient to restore the cognitive impairment observed in gp120tg mice. Our data suggest that activation of p75NTR is one of the mechanisms crucial for the neurotoxic effect of gp120. These data indicate that p75NTR antagonists could provide an adjunct therapy against synaptic simplification caused by HIV.

## Introduction

Despite the use of combination antiretroviral therapy (cART) (Ellis et al., 2007; Everall et al., 2009), approximately half of HIV-positive individuals are at a high risk for developing mild to severe cognitive impairments, termed HIV-associated neurocognitive disorders (HANDs) (Clifford and Ances, 2013; Saylor et al., 2016). Cognitive alterations seen in HAND subjects correlate with loss of synapses (Masliah et al., 1997; Albright et al., 2003; McArthur, 2004; Everall et al., 2005; Crews et al., 2009). However, our understanding of the mechanisms of HIV-mediated synaptic degeneration is incomplete. A better understanding of the molecular mechanisms underlying HIV neurotoxicity could lead to a new adjunct therapy for HIV positive individuals.

The brain serves as a reservoir for ongoing HIV replication (Fois and Brew, 2015); in fact, HAND subjects have detectable levels of HIV RNA in their cerebrospinal fluid (CSF) even when the virus is undetectable in the blood (Di Carlofelice et al., 2018). However, HIV does not infect neurons and thus HAND must result from mechanisms other than neuronal infection. HIV may evoke neuronal injury through indirect mechanisms such as neurotoxins released by infected or immune-stimulated, inflammatory microglia and macrophages (Kaul et al., 2001). Neuronal injury may also result from neurotoxic action of viral proteins such as the activator of transcription Tat (Nath and Steiner, 2013) or the envelope protein gp120 (Meucci and Miller, 1996). The molecular mechanisms whereby gp120 promotes synaptic simplification are still under investigation. The loss of neurons, simplification of neuronal branching, and reduction in dendritic spines can also be triggered by the p75 neurotrophin receptor (p75NTR) (reviewed in Ibanez and Simi, 2012), a member of the tumor necrosis factor receptor family which contains a death domain (Feinstein et al., 1995; Liepinsh et al., 1997). Indeed, activation of p75NTR induces neuronal cell death (Teng et al., 2005) as well as axonal and dendritic spine pruning both during development (Singh et al., 2008) as well as in the adult nervous system (Park et al., 2010; Kraemer et al., 2014).

There are many ligands for the p75NTR. These include mature as well as unprocessed neurotrophins or proneurotrophins (Chao, 2003), myelin-associated glycoproteins (Wong et al., 2002) and beta amyloid peptide (Perini et al., 2002; Knowles et al., 2013). A p75NTR ligand that promotes neuronal apoptosis and synaptic pruning is the proneurotrophin brain-derived neurotrophic factor (proBDNF) (Pang et al., 2004; Teng et al., 2005; Yang et al., 2014; Guo et al., 2016). Previous work from our laboratory has shown that gp120 increases the levels and release of proBDNF in primary neuronal cultures (Bachis et al., 2012). In these cultures, p75NTR inhibitors block gp120-mediated synaptic simplification (Bachis et al., 2012), suggesting that activation of p75NTR by proBDNF may be a crucial mechanism to underlying the synaptic simplification seen in HAND. This suggestion is supported by evidence showing that postmortem brains of HAND subjects exhibit higher levels of proBDNF than HIV positive subjects without cognitive alterations (Bachis et al., 2012). Consistently with this suggestion, recent data have shown that increased hippocampal proBDNF contributes to memory impairment in aged mice (Buhusi et al., 2017).

This present study was undertaken to provide molecular and behavioral evidence of the role that p75NTR plays in gp120-mediated loss of synaptic contacts. We utilized gp120 transgenic (gp120tg) mice intercrossed with *p75NTR* null mice. The gp120tg mice display a multitude of altered neuron-specific processes, including synaptic simplifications (Toggas et al., 1994; Bachis et al., 2016b) and impaired neurogenesis (Lee et al., 2011), as well as cognitive deficits (D’Hooge et al., 1999) and sensorimotor gating impairments (Henry et al., 2014), suggesting that these animals are a suitable model to study HAND (Thaney et al., 2018). We report that the reduction of *p75NT*R expression significantly decreases the neurotoxic effect of gp120 as well as impairment in memory evoked by gp120.

## Materials and Methods

### Reagents

Human T-lymphotropic virus (HTLV)-IIIB (HIV1_*IIIB*_) was obtained through the AIDS Research and Reference Reagent Program, Division of AIDS, National Institute of Allergy and Infectious Diseases (NIAID), National Institutes of Health (NIH). Gp120IIIB was obtained for Immunodiagnostics, Inc. (Woburn, MA, United States). Uncleavable proBDNF was purchased from Alomone labs (Jerusalem, Israel).

### Cortical Neurons

Primary mouse cortical neurons were prepared from the cortex of embryonic wild type after days 17–18 (WT) and *p75NTR*^–^*^/^*^–^ mice following an established protocol (Avdoshina et al., 2016a, b). Cells were seeded (0.5 × 10^6^/ml) onto poly-L-lysine (Sigma Aldrich) pre-coated plates or glass coverslips in Neurobasal Medium containing 2% B27 supplement, 25 nM glutamate, 0.5 mM L-glutamine, and 1% antibiotic-antimycotic solution (Thermo Fisher Scientific). Cultures were grown at 37°C in 5% CO_2_/95% air for 7 days prior to the experiments. At day 7 *in vitro*, cell cultures contained 95% neurons as characterized by an antibody against tubulin β III (TUBB3), as previously described (Avdoshina et al., 2016a, b).

### Cell Viability

The viability of primary cortical neurons was estimated by Hoechst 33258 and propidium iodide (Hoechst/PI; Sigma-Adrich) co-staining and visualized using a fluorescence microscope Olympus IX71, as previously described (Avdoshina et al., 2016a). Hoechst/PI-positive cells were then counted using ImageJ (National Institutes of Health, Bethesda, MD, United States) and expressed as a percentage of the total number of neurons.

### Animals

Gp120tg breeding mice were obtained from Dr. E. Masliah (University of California, San Diego, San Diego, CA, United States). The characterization of these mice is provided elsewhere (Toggas et al., 1994). Female gp120tg mice were intercrossed with C57BL/6J male *p75NTR*^–/–^ mice (The Jackson Laboratory, Bar Harbor, ME, United States) to generate males and female *p75*^–/–^ and *p75*^+/–^gp120tg mice, as previously described (Bachis et al., 2016b). Wild type (WT) littermates (gp120 null/*p75*^+/+^) were generated from these colonies and used as controls for our biochemical, behavioral, and histological studies. Animals were housed under standard conditions with food and water *ad libitum* and maintained on a 12-h light/dark cycle. Mice were maintained in our facility for up to 10 months. 8–10 month old mice (of both sexes) were used for these studies. An animal’s genotype was confirmed through an outsourced genotyping service (Transnetyx, Inc., Cordova, TN, United States) from tail snips taken at time of weaning and at sacrifice. All studies were carried out following the Guide for the Care and Use of Laboratory Animals as adopted and promulgated by the U.S. National Institutes of Health and approved by the Georgetown University Animal Care and Use Committee.

### Behavioral Analysis

All rodents in this study were tested during their dark (active) period. For each behavioral test, mice were brought to the testing room and allowed to habituate to the testing conditions for at least 1 h. White noise (50 dB) was played to obscure noises from outside the testing room. After the conclusion of each test, mice were returned to their home cages in the animal facility. The assays were scheduled in an order to minimize the impact of repeated testing on performance and occurred in the same order as they appear below within section “Materials and Methods.”

#### Open Field Measures

The open field apparatus (Med Associates, Inc., Saint Albans City, VT, United States) measured 27 cm × 27 cm and had transparent walls of 20 cm. The apparatus also contained 16-beam IR arrays on both the X and Y axes for positional tracking within the apparatus and on the Z axis for rearing detection. In order to encourage exploration, the open field was dimly lit by overhead room lights at 75 lux. The apparatus was cleaned with a 70% ethanol solution between trials. Mice were placed in the center of the field and exploration was recorded over a single trial of 60 min. Behavior was tracked through the IR beam array and analyzed by the Med Associates Activity Monitor software. The rodents’ behavior in this apparatus was analyzed using IR beam breaks for locomotor activity throughout the trial. The center zone was defined as the zone greater than 6 cm from any of the walls.

#### Passive Avoidance

The modular passive avoidance chamber (Coulbourn Instruments, Holliston, MA, United States) had two enclosed chambers of equal dimensions separated by a wall. This center wall had a 6 cm by 6 cm guillotine door linked to a computer-controlled AMi-2 interface device (Stoelting, Co., Wood Dale, IL, United States). Each chamber in the apparatus measured 17.0 cm by 17.7 cm and had a height of 30.5 cm. One side of the chamber had opaque walls and provided a dark environment for rodents inside this compartment. The other compartment was brightly illuminated by an overhead light at 300 lux. The chamber was placed in the center of the room with indirect overhead lighting and a side-mounted remote USB camera for viewing mice within the apparatus.

The passive avoidance task was conducted over three consecutive days with a single trial on each day (Day 1: habituation, Day 2: acquisition, Day 3: retention probe trial). In the habituation trial, mice were placed in the lighted chamber with the door closed and allowed to explore for 180 s. For the acquisition trial, mice were again placed in the lit compartment at the beginning of the test. After 30 s, the door lifted and mice were given access to the dark compartment. When a mouse had entered the dark compartment, the experimenter closed the door with a remote switch, and a computer program (Anymaze, Stoelting, Co., Wood Dale, IL, United States) initiated a 2 s foot shock at 0.2–0.4 mA. After five additional seconds in the dark compartment, the test was ended and the mouse was retrieved. The probe trial followed an identical procedure to the acquisition trial, but the door was closed and the mouse was not shocked when it entered the dark zone. The probe trial was limited to 300 s. If the mouse had not entered within 300 s, the mouse was removed from the apparatus and its probe latency was recorded as 300 s. The latency to enter the dark zone on the acquisition and probe trials was recorded by the Anymaze software via a keystroke from the experimenter. A mouse was judged to have entered the dark compartment when all four paws were completely inside the darkened chamber.

#### Morris Water Maze

The Morris water maze (MWM) apparatus consisted of a circular pool (120 cm in diameter), which was filled to a depth of 50 cm with 26°C water. Habituation, acquisition, and reversal trials included a 6 cm by 6 cm escape platform submerged ∼1 cm below the water’s surface. The maze was lit by overhead lights at 75 lux and surrounded by white curtains with large distal cues on each of the four cardinal directions.

We conducted our MWM paradigm over 13 consecutive days. Briefly, rodents were given a single 60 s habituation trial in clear water with a submerged, but visible escape platform before training began. Spatial acquisition trials were performed four times per day and conducted over the next 5 days with the water now made opaque by the addition of white acrylic paint. A single probe trial was performed 24 h after the final spatial acquisition trial with the escape platform now absent from the maze. Reversal trials on the next 5 days were conducted in a manner identical to the spatial acquisition trials, but with the escape platform moved 180° to its initial position within the apparatus. Finally, the reversal probe trial was conducted on the final day in a manner identical to the initial probe trial. The habituation trial and probe trials were limited to 60 s. The acquisition and reversal trials were likewise limited to 60 s, but mice were gently guided to the escape platform if they had not located this platform within 60 s. Animals were allowed to remain on the escape platform at the end of their trial for 15 s in order to examine their location with respect to the distal cues. We used an inter-trial interval of 15 min.

The MWM was virtually divided into four equal quadrants and behavior was analyzed by Anymaze for latency to entry onto the escape platform, duration in target quadrant, duration in the center (non-thigmotaxic) area, passes over the former escape platform location, and average swimming speed. A trial was excluded from MWM analysis if the animal demonstrated non-searching behaviors in the maze, which we defined *a priori* as passive floating for greater than five consecutive seconds or panicked swimming at one location on the maze wall (less than 2% of all trials). An animal was omitted from a testing day if two or more trials were excluded within the same day, but every mouse was allowed to finish the trial and remain on the target platform for each exposure to the MWM.

### Preparation of Synaptosomes and Western Blot Analysis

Mice were euthanized by cervical dislocation for the preparation of synaptosomes. Synaptosomes were prepared from brain lysates using Synaptic Protein Extraction Reagent (Thermo Fisher Scientific, Inc., Waltham, MA, United States) according to the manufacturer instructions. Protein content was determined by BCA Protein Assay Reagent Kit (Thermo Fisher Scientific, Inc.) according to the manufacture instructions. Proteins were separated in a NuPAGE 4–12% Bis-Tris Gel and transferred to a nitrocellulose membrane using iBlot device (Thermo Fisher Scientific, Inc.). Membranes were blocked with 5% milk in PBS and 0.1% Tween-20 and probed with antibodies against: PSD95 (1:2000, Thermo Fisher Scientific, Inc.), NMDAR2B (1:1000, Abcam, Inc., Cambridge, United Kingdom), NMDAR2A (1:1000, R&D Systems, Minneapolis, MN, United States), and synaptophysin (1:2000, Sigma-Aldrich, Co., St. Louis, MO, United States). Membranes were stripped with Restore Western Blot Stripping Buffer (Invitrogen) for 30 min at 37°C and re-probed with and anti-β-actin antibody (1:15000, Sigma-Aldrich, Co.) in blocking buffer to serve as a protein loading control. Immune complexes were detected with the corresponding secondary antibody and chemiluminescence reagent (Fisher Scientific). The intensity of immunoreactive bands was quantified using ImageJ and expressed in arbitrary units (AUs) defined as optical densities of synaptic protein relative to β-actin.

### Statistical Analysis

Data, expressed as the mean ± SEM, were analyzed using one or two-way analysis of variance (ANOVA) with either Tukey’s HSD *post hoc* test for biochemistry, or Kruskal–Wallis for behavior, using GraphPad Prism software v. 7.0 (GraphPad). A *p*-value < 0.05 was considered statistically significant.

## Results

### gp120 Is Not Neurotoxic in *p75NTR*^–/–^ Neurons

**FIGURE 1:**
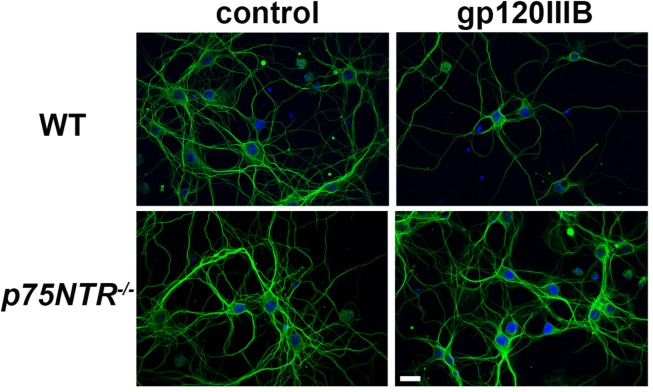
gp120 does not cause neurite pruning in *p75NTR*^– /–^ neurons. Representative images of primary neuronal cultures prepared from E17 cortex of WT or p75NTR^– /–^ mice. At 7 days in culture, neurons were exposed to heat inactivated (boiled) gp120IIIB (control) or 5 nM gp120IIIB for 6 h. Cells were fixed and processes visualized by anti TUBB3 antibody (green). DAPI (blue) was used to stain nuclei. Bar = 20 μm. Note that the neuronal network (TUBB3 positive processes) in p75NTR^– /–^ neurons exposed to gp120 is preserved when compared to WT neurons exposed to gp120. The experiment was repeated twice.

We have previously demonstrated that gp120, which induces the releases proBDNF, promotes synaptic pruning in rodent primary neurons (Bachis et al., 2012; Avdoshina et al., 2017). The neurotoxic effect of gp120 is prevented by p75NTR antagonists (Bachis et al., 2012). To further support these data, gp120 was applied to primary cultures of cortical neurons obtained from WT or *p75NTR*^–^*^/^*^–^ mice for 6 h and neuronal processes were identified by an antibody against neuron-specific cytoskeleton protein tubulin β III (TUBB3). Consistent with our prior findings (Bachis et al., 2012), exposure of neurons from WT mice to gp120 (5 nM) reduced the overall TUBB3 immunoreactivity, suggesting a decrease in the number of neuronal processes (Figure 1). Importantly, we found that neurons lacking *p75NTR* expression have a more complex TUBB3-positive network than neurons from WT animals exposed to gp120 (Figure 1).

**FIGURE 2:**
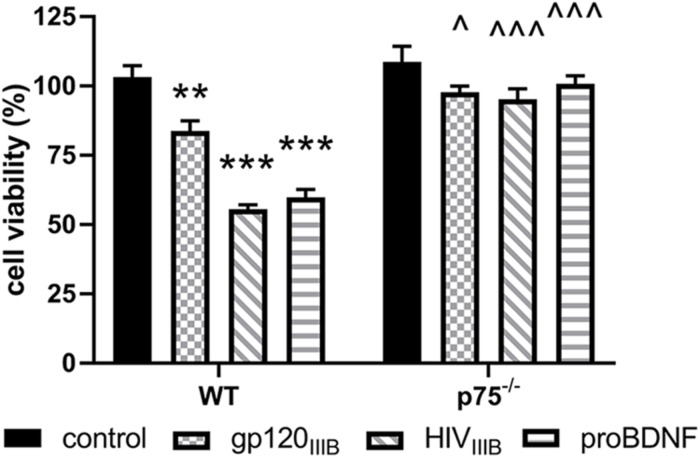
The neurotoxic effect of HIV and gp120 is reduced in *p75NTR*^– /–^ neurons. Cortical neurons from WT and p75NTR^– /–^ mice were exposed to gp120_*IIIB*_ (5 nM), HIV_*IIIB*_ (1.5 ng/ml of p24), or proBDNF (10 nM) for 24 h. Boiled inactivated gp120 or HIV (IIIB) were used as controls. Neuronal cell death was determined by counting the number of Hoechst/PI positive cells, as described in Section “Materials and Methods.” Data expressed as % of cell viability are the mean ± SEM of eight independent coverslips from four independent culture preparations; coverslip values were averaged from three random fields. ^∗∗^*p* < 0.01, ^∗∗∗^*p* < 0.001 vs. WT control, ^*p* < 0.05 vs. WT gp120_*IIIB*_, ^ ^ ^*p* < 0.01 vs. WT HIV_*IIIB*_ or proBDNF. Two-way ANOVA and Tukey’s HSD.

To provide a quantitative assessment of the neurotoxic effect of gp120, we exposed WT and p75NTR^–/–^ neurons for 24 h to gp120 (5 nM). Hoechst/PI was used to quantify the number of surviving neurons. Furthermore, we examined whether HIV, which shares a similar neurotoxic profile of gp120 in rodent neurons (Bachis et al., 2009), is neurotoxic via p75NTR. As a positive control for p75NTR-mediated loss of neurons, we also exposed both WT and *p75NTR* null neurons to proBDNF for 24 h (10 nM). WT neurons in the presence of gp120, HIV or proBDNF displayed the expected increase in the number of neurons with Hoechst/PI staining, indicating increased apoptosis (Figure 2); importantly, the lack of *p75NTR* significantly reduced neuronal loss caused by either gp120, HIV, or proBDNF (Figure 2). Overall, a two-way ANOVA for this set of experiments revealed significance for genotype (*F*_(__1__,__60__)_ = 91.92; *p* < 0.001), treatment (*F*_(__3__,__60__)_ = 27.71; *p* < 0.001), and interaction (*F*_(__3__,__60__)_ = 12.88; *p* < 0.001) factors. Taken together, our data suggest that p75NTR mediates the synaptic pruning effect of gp120, most likely shed from the virus.

### gp120 Decreases PSD95 and NMDA Receptor Subunit Immunoreactivity

We have previously shown that gp120 causes a decrease in the number of dendritic spines in the hippocampus, an effect that is significantly diminished by the removal of one *p75NTR* allele (Bachis et al., 2016b). Dendritic spines form the post-synaptic density of the majority of excitatory synapses. Thus, to determine whether gp120 affects post-synaptic spines, we prepared synaptic fractions from homogenized mouse brains of 8–10 month-old WT and gp120tg mice and measured the levels of post-synaptic and presynaptic proteins. These include post-synaptic density protein 95 (PSD95), an abundant scaffolding protein that determines the functional integrity of excitatory synapses, *N*-methyl-D-aspartate (NMDA) receptor (NR) subunit 2A and 2B (Kornau et al., 1995) and synaptophysin, a transmembrane protein that is involved in synaptic formation and exocytosis. We first verified the appropriateness of the method by determining PSD95 and NR2A and 2B subunits in brain lysates from WT mice containing synaptosomal and cytoplasmic preparation. Data shown in Supplementary Figure S1 confirm that PSD95, NR2A, and 2B immunoreactivity are only found in synaptosomal preparations. We then examined whether the levels of these synaptic proteins are altered in the hippocampus of gp120tg mice.

**FIGURE 3:**
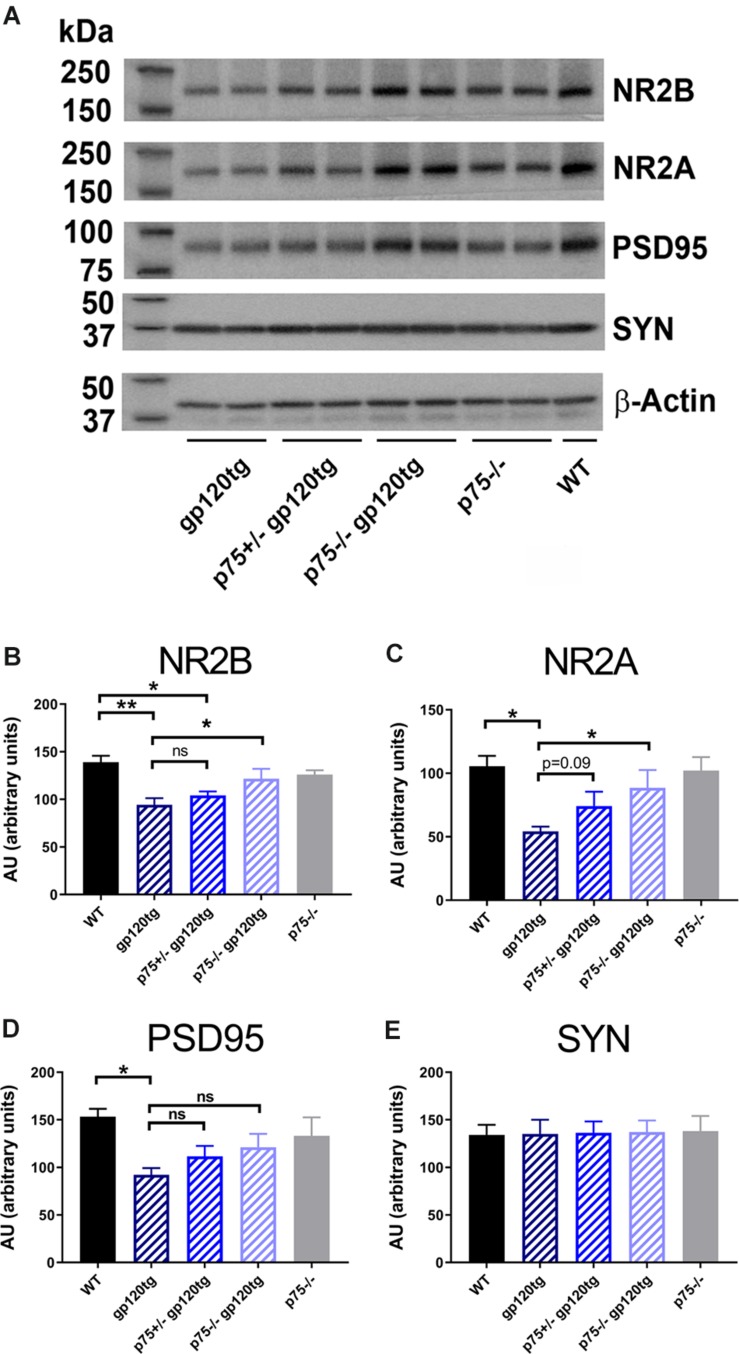
Analysis of synaptosomal preparations from gp120 and *p75NTR* mice. **(A)** Example of a Western blot analysis of synaptosomal preparation from the hippocampus of the indicated mouse genotypes. The blot was probed with antibodies that recognized the indicated synaptic proteins. Beta-actin was used for loading control. Molecular weights of analyzed proteins are listed on the left. **(B–E)** Levels of synaptic proteins, quantified as described in Section “Materials and Methods” and expressed in AU, are the mean ± SEM of five animal per group. ^∗^*p* < 0.05, ^∗∗^*p* < 0.01. In all panels, comparisons of means of WT and p75^– /–^ mice reveal no significant differences. ANOVA and Tukey’s HSD.

When compared to WT, hippocampal synaptosomes from gp120tg mice exhibited a decrease in PSD95, NR2A and 2B subunits (Figure 3A). In fact, one-way ANOVAs for synaptosomal contents of PSD95 (*F*_(__4__,__19__)_ = 3.674; *p* < 0.05), NR2A (*F*_(__4__,__18__)_ = 4.101; *p* < 0.05), and NR2B (*F*_(__4__,__19__)_ = 6.395; *p* < 0.01) showed significant differences among means. Interestingly, gp120 did not change the levels of synaptophysin (Figure 3E) (*F*_(__4__,__19__)_ = 0.01391; *p* > 0.9996). Thus, it appears that gp120 may target mainly post-synaptic densities. Importantly, the removal of *p75NTR* mitigated the effect of gp120 (Figure 3). It is important to note that lack of *p75NTR* expression *per se* did not change the levels of these synaptic markers when compared to WT mice (Figure 3), supporting a previous study showing that removal of p75NTR *in vivo* does not induce abnormal alteration of synapses (Qian et al., 2018).

### gp120-Mediated Deficits in Performance on a Passive Avoidance Task Is Inhibited by the Removal of *p75NTR* Alleles

Gp120tg mice develop age-related cognitive abnormalities, which correlate with loss of synaptic plasticity and neuronal degeneration (Toggas et al., 1994; Krucker et al., 1998; Lee et al., 2011), as well as an increased in the levels of proBDNF in the hippocampus (Bachis et al., 2016b). These data allowed us to speculate that a reduction of *p75NTR* expression would avert the impaired performance on hippocampal-dependent memory tasks previously described in gp120tg mice (Krucker et al., 1998; D’Hooge et al., 1999).

**FIGURE 4:**
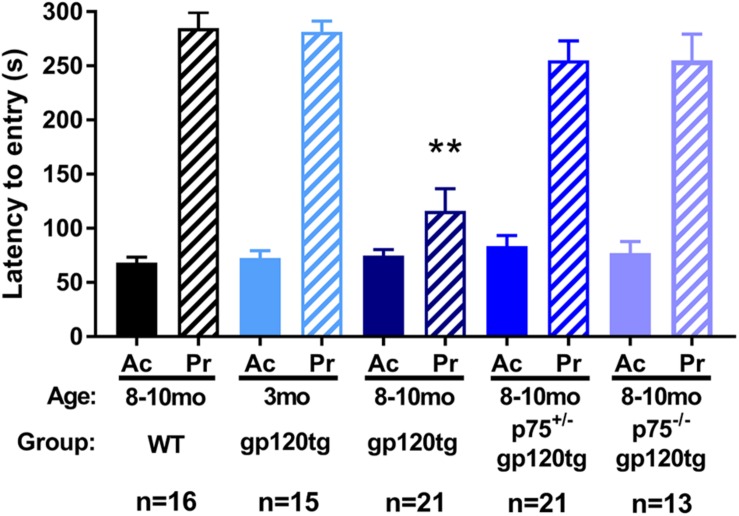
Removal of *p75NTR* rescues gp120-driven impairments on a passive avoidance task in (8–10mo) animals. 3- (3mo) and 8–10 month (8mo)-old mice of the indicated genotypes were subjected to a passive avoidance task to evaluate impairments in avoidance earning. Latency to enter the dark compartment on acquisition (Ac) and probe (Pr) testing days was analyzed. ANOVA with Tukey’s HSD for comparisons across genotypes. ^∗∗^*p* < 0.001 vs. WT. Data are expressed as mean ± SEM. *n* = number of animals per group.

To examine whether loss of hippocampal spines was associated with impaired long-term avoidance memory, we subjected 8–10 month-old WT, gp120tg, p75^+/–^gp120tg, and p75^–/–^gp120tg mice to a passive avoidance task. Mice of each genotype entered the dark compartment with similar latency on the acquisition trial (one-way ANOVA: *F*_(__4__,__81__)_ = 0.5615, *p* = 0.6912) (Figure 4), suggesting that the absence of one or both *p75NTR* alleles does not affect exploratory drive in this apparatus. Across all probe trials, gp120tg mice showed a significant difference in latency to enter the dark compartment compared to WT. The gp120-mediated impairment in passive avoidance was reduced in gp120tg mice with one or both *p75NTR* alleles missing (Kruskal–Wallis with *post hoc* Dunn’s test *H* = 30.14, df = 4, *p* < 0.001) (Figure 4). Interestingly, 3 month-old (3mo) gp120tg mice performed better than 8–10 month-old (8–10mo) gp120tg mice, supporting previous data that gp120-induced behavioral effects are age dependent (Toggas et al., 1994; D’Hooge et al., 1999; Bachis et al., 2016a). In fact, two-tailed Wilcoxon matched-pairs signed-rank tests performed between the acquisition and probe testing days within each genotype shows significant differences within 8–10mo WT (*p* < 0.001), 3mo gp120tg (*p* < 0.001), 8–10mo p75^+/–^gp120tg (*p* < 0.001), and 8–10mo p75^–/–^gp120tg (*p* < 0.001) groups, but not within 8mo gp120tg (*p* = 0.0973).

**FIGURE 5:**
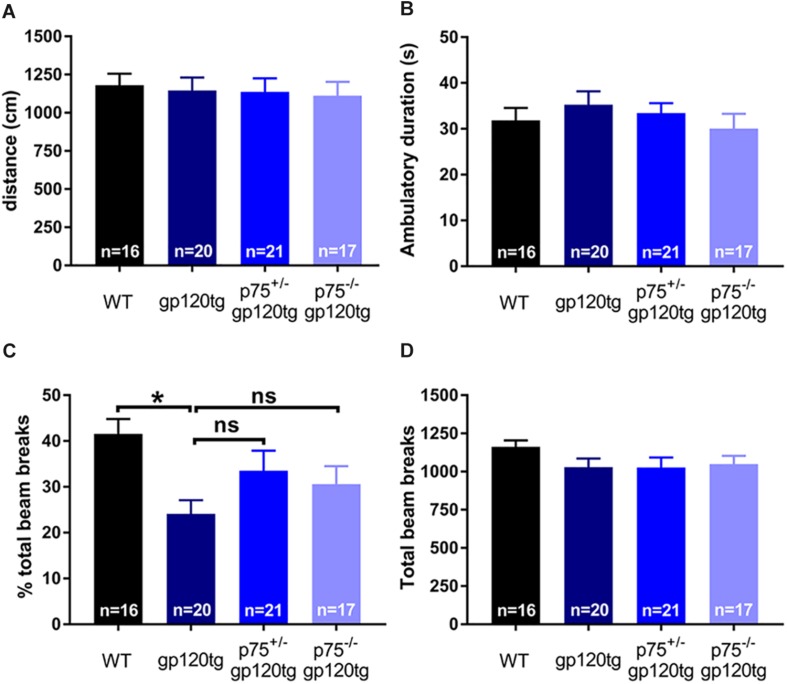
Deletion of one or both *p75NTR* alleles in gp120 mice does not affect locomotion or exploration in a novel environment. 8–10 month-old mice were exposed for 5 min to an open field. **(A)** Cumulative distance traveled in the open field. **(B)** Total time spent ambulating in the open field. **(C)** Percent of total beam breaks occurring in the center of the open field. ANOVA with Tukey’s HSD. ^∗^*p* < 0.05 vs. WT. **(D)** Total active beam breaks (ambulatory and stereotypic movements) occurring within the 5-min trial. Data are expressed as mean ± SEM. *n* = number of animals per group.

The observed effect may be due in part to differential locomotor activity, vigilance, or alertness in a novel environment among experimental groups (i.e., more exploratory animals may enter the dark compartment at a greater rate regardless of a formed association). To address these confounds, we assessed the above behaviors in a single 5-min exposure to an open field. Both the cumulative distance traveled (Figure 5A) and the time spent ambulating (Figure 5B) in the trial were equivalent between experimental groups (one-way ANOVA: *F*_(__3__,__70__)_ = 0.0973, *p* = 0.9613, *F*_(__3__,__69__)_ = 0.6548, *p* = 0.5827, respectively). The percentage of total active beam breaks occurring in the center of open field was decreased in 8–10mo gp120tg mice when compared to WT, replicating previous findings that older gp120tg mice display a modest anxious phenotype (one-way ANOVA: *F*_(__3__,__69__)_ = 3.748, *p* = 0.0148) (Henry et al., 2014; Bachis et al., 2016a). Interestingly, this effect was rescued in animals by deleting one or both *p75NTR* alleles (Figure 5C). However, the total active beam breaks (Figure 5D) were equivalent across experimental groups (one-way ANOVA: *F*_(__3__,__71__)_ = 1.163, *p* = 0.3300). Thus, although gp120tg mice show modest anxiety-like behaviors in a novel environment, all groups have a comparable exploratory drive and activity level in a novel environment within short passive avoidance timeframes. Based on these data, it is unlikely that the deficits seen in the passive avoidance task arise from anxiousness in an open environment.

### Genetic Deletion of *p75NTR* Rescues gp120-Mediated Impairment in Spatial Memory

**FIGURE 6:**
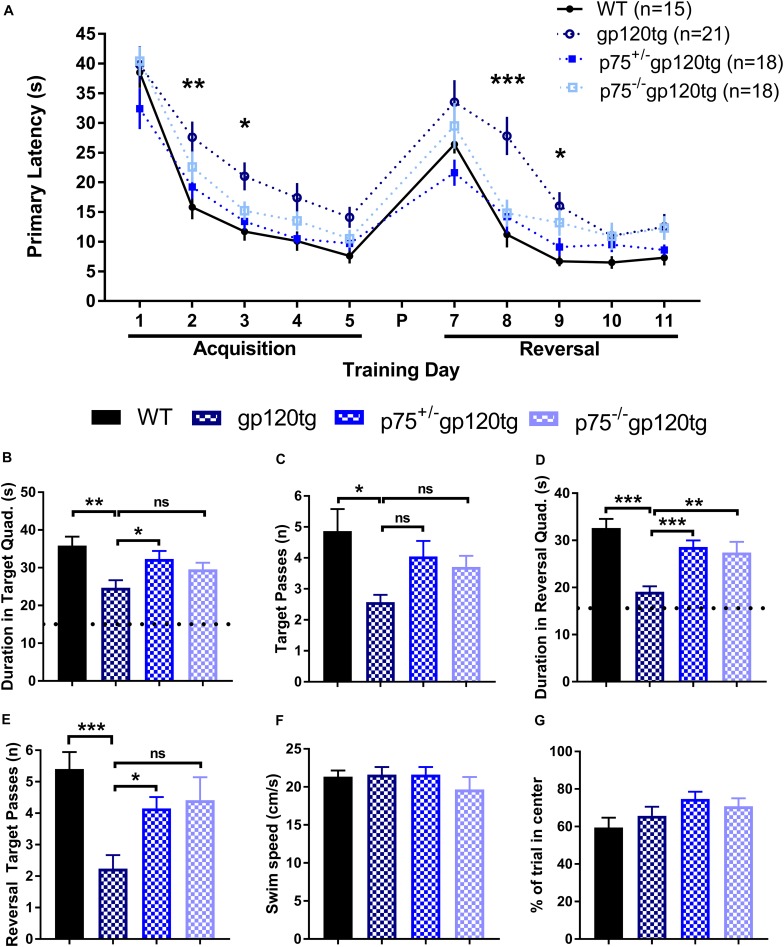
gp120tg mice show impairments in a task of spatial navigation. 8–10 month-old mice were evaluated with the Morris water maze (MWM) navigation task. **(A)** Latency to locate the submerged escape platform in the maze over five training days of acquisition and five days of reversal learning. These two learning phases were separated by a probe (P) on day 6 and a reversal probe on day 12. One-way ANOVA (within each TD) with Tukey’s HSD. Each data point represents the genotype mean of the average of an animal’s trials on a training day. **(B)** Duration in the target quadrant during the first probe trial. Dotted line indicates chance performance. One-way ANOVA with Tukey’s HSD. **(C)** Passes over the former location of the target platform during the probe trial. Kruskal–Wallis with *post hoc* Dunn’s. **(D)** Duration in the reversal target quadrant during the first probe trial. Dotted line indicates chance performance. One-way ANOVA with Tukey’s HSD. **(E)** Passes over the former reversal escape during the reversal probe trial. Kruskal–Wallis with *post hoc* Dunn’s. **(F)** Habituation swim speed and **(G)** percent of first trial in the center of the MWM were taken as control measures for locomotion and motivation to explore the maze, respectively. One-way ANOVA. ^∗^*p* < 0.05, ^∗∗^*p* < 0.01, ^∗∗∗^*p* < 0.001. Data are displayed as mean ± SEM. *n* = number of animals per group.

Previous studies have demonstrated that spatial memory is impaired in gp120tg mice (D’Hooge et al., 1999). We hypothesized that spatial learning and memory, a hippocampal-dependent behavior, would be improved in mice lacking one or both *p75NTR* alleles. To assess impairments in spatial memory, we employed a MWM navigation task over 13 days. WT mice performed significantly better than gp120tg mice in both the acquisition and reversal phase, in which the escape platform was moved 180° from its original location. Indeed, the gp120tg mice showed impairments on the second and third acquisition and reversal days (Figure 6A). The removal of one or both *p75NTR* alleles diminished the effect of gp120. Supplementary Table S1 displays all statistical measures and inter-group comparisons within the two MWM learning phases. A probe trial was administered 24 h after both the final acquisition and reversal trials. These probes revealed differences between gp120tg mice vs. WT controls with respect to the duration of time spent in the target quadrant (Figure 6B) and passes over the former target platform’s location (Figure 6C). Both p75^+/–^gp120tg and p75^–/–^gp120tg groups had non-significant differences in these two probe measures vs. WT controls. Similar results were observed within the reversal probe (Figures 6D,E, respectively).

To account for possible confounds due to impairments in swimming, we compared swim speeds during the initial habituation to the water maze. Swim speed did not differ significantly (one-way ANOVA: *F*_(__3__,__71__)_ = 0.6611, *p* = 0.5787) between the four genotypes during this single trial (Figure 6F). Likewise, the percentage of time spent swimming in the center of the MWM on the first trial following habituation was similarly equivalent across experimental groups (Figure 6G; one-way ANOVA: *F*_(__3__,__71__)_ = 1.964, *p* = 0.1271), indicating comparable motivation to escape the maze. Taken together, these data indicate that there are differences in spatial learning and memory across genotypes.

## Discussion

Dendritic injury and synaptic dysfunction are believed to cause the cognitive decline in HAND and other neurodegenerative diseases. Loss of synapses, similar to what is seen in HAND, is also reproducible in transgenic mice overexpressing gp120 (reviewed in Thaney et al., 2018). In this work we have used this animal model to characterize molecular/cellular mechanisms underlying the neuropathology of HAND. Our previous studies have shown that gp120tg mice exhibit increased levels of proBDNF in the hippocampus (Bachis et al., 2016b). Moreover, gp120 induces the release of proBDNF from neuronal cultures (Bachis et al., 2012). Here, we show that gp120 neurotoxicity can be attenuated by the removal of p75NTR, a receptor that promotes synaptic pruning (Zagrebelsky et al., 2005; Singh et al., 2008) and neuronal cell death (Bamji et al., 1998; Bhakar et al., 2003). Thus, our results support the suggestion that gp120 promotes synaptodendritic injury by a mechanism that favors the activation of p75NTR.

How can gp120 neurotoxicity be linked to p75NTR activation? ProBDNF, like other proneurotrophins, is cleaved into mature BDNF in the endoplasmic reticulum by the proconvertase furin (Seidah et al., 1996) or extracellularly by proteases such as plasmin and matrix metalloproteases (Pang et al., 2004). Gp120 decreases the level and activity of furin and plasmin, thus reduces the conversion of proBDNF to mature BDNF (Bachis et al., 2012). Consequently, gp120tg mice exhibit higher levels of proBDNF than WT in the hippocampus and other brain areas (Bachis et al., 2016b). Moreover, gp120 promotes the release of proBDNF from cortical neurons and alters the ratio mature BDNF/proBDNF in the synaptic cleft in favor of proBDNF. This release could compromise synaptic connections and neuronal survival as indicated by the increased neuronal loss in cortical neurons exposed to gp120 (Bachis et al., 2012). Our data obtained in p75NTR^–/–^ neurons, in which the neurotoxic effect of gp120 was significantly attenuated, strongly suggest that gp120-mediated synaptodendritic injury and cell loss depend upon an indirect activation of p75NTR.

The number and morphology of dendritic spines have emerged as crucial components underlying synaptic plasticity. Dendritic spines express all ionotropic glutamatergic receptors, which play a central role in long-term potentiation (LTP) (Kasai et al., 2010; Rochefort and Konnerth, 2012), a well-studied form of synaptic plasticity that forms the cellular basis of hippocampal-dependent learning and memory (Herron et al., 1986; O’Dell et al., 1991). Gp120 has been shown to inhibit LTP (Sanchez-Alavez et al., 2000; Dong and Xiong, 2006), which would be consistent with a reduced spine density in the hippocampus described in gp120tg mice (Bachis et al., 2016b). In this study, we have provided preliminary but complimentary data showing that gp120 decreases the levels of NR2A and 2B subunits. This decrease is particularly important because these subunits play a role in glutamate-mediated synaptic plasticity (Liu et al., 2004; von Engelhardt et al., 2008). Moreover, both PSD95 and NR subunits, are considered markers for excitatory post-synaptic sites (Sheng, 2001). Intriguingly, gp120 failed to change the levels of synaptophysin, a synaptic vesicle membrane protein found predominantly presynaptically (Tarsa and Goda, 2002). Thus, it appears that gp120 may target mostly the post-synaptic membrane. This suggestion, although still speculative, is in line with the fact that neurons release proBDNF (Yang et al., 2009), which then acts on post-synaptic p75NTR to decreases spine density of hippocampal pyramidal neurons (Zagrebelsky et al., 2005; Yang et al., 2014).

Cognitive impairment and reduced LTP seen in gp120 mice (Krucker et al., 1998; D’Hooge et al., 1999) correlate with loss of synapses in the hippocampus (Toggas et al., 1994; Lee et al., 2013; Bachis et al., 2016b). These effects appear when mice are at least 6 months old. In the present study, we have used a series of behavioral tests that assess loss of hippocampal connections to determine whether memory impairment in gp120tg mice could be abolished by the removal of *p75NTR* alleles. We have found that the hippocampal-dependent memory deficits observed in 8–10mo gp120tg mice is attenuated when either one or both *p75NTR* alleles are removed. Thus, reduced expression of *p75NTR*, which has been shown to slow down cognitive decline in an animal model of Alzheimer’s disease (Qian et al., 2018), not only inhibits the loss of hippocampal spines that we have previously described (Bachis et al., 2016b) but also precludes the impairment in memory observed in 8–10mo gp120tg mice. In addition, we observed that the removal of *p75NTR* alleles reduces the impairments seen in the MWM reversal phase in gp120tg mice. Reversal learning has multiple neural substrates in rodents independent of the hippocampus, including the subnuclei of the basal forebrain and the prefrontal cortex (Ghods-Sharifi et al., 2008; Tait and Brown, 2008). Although we cannot exclude that the ability of gp120 to increase proBDNF in any of these areas may underlie a reversal learning impairment, it is difficult to interpret reversal learning deficits when impairments in general spatial learning are also seen within the MWM test. Therefore, we exert caution in interpretation of this curious finding and recognize that more rigorous assays of reversal learning are needed in future studies with this specific model of HAND.

The mechanism(s) whereby proBDNF activation of the p75NTR reduces spine density remains to be established. p75NTR, after binding to sortilin family member SorCS2, activates several signaling pathways that are crucial for neuronal degeneration. These include c-Jun N-terminal kinase (JNK) (Friedman, 2000; Salehi et al., 2002), the RhoA (Park et al., 2010) and the NF-kB pathways (Carter et al., 1996). JNK is also activated by the HIV protein gp120 (Meucci et al., 1998; Bodner et al., 2004; Singh et al., 2005), suggesting a common neurotoxic mechanism between viral proteins and p75NTR. Experimental studies have also shown that p75NTR, destabilizes actin filaments through inactivation of Rac/fascin interaction (Deinhardt et al., 2011). Actin influences spine morphology and stability (Rust et al., 2010). Moreover, p75NTR has been shown to inhibit neurite outgrowth by interacting with the Nogo receptor complex (Barker, 2004) and Ephrin-A (Lim et al., 2008), important components of synapses and promoters of spine morphogenesis (Lai and Ip, 2009). On the other hand, we need to consider that the hippocampus of gp120tg mice as well as HAND subjects exhibits lower levels of BDNF than controls (Bachis et al., 2012, 2016b). BDNF has been shown to promote maturation and density of dendritic spines (Orefice et al., 2013). Thus, a reduction in BDNF levels in favor to proBDNF levels, as seen in gp120tg mice and HAND subjects, may accelerate synaptic pruning. Moreover, we cannot exclude that gp120-mediated synaptic pruning is linked to the ability of the envelope protein to decrease the levels of BDNF receptor trkB (Bachis et al., 2016b). This receptor modulates synaptic plasticity and spine density in the adult hippocampus (Yacoubian and Lo, 2000; Otal et al., 2005), as well as participates in spine maintenance (Chapleau and Pozzo-Miller, 2012). Higher proBDNF and lower trkB levels have been discovered in the postmortem hippocampus of HAND subjects compared to non-cognitive impaired HIV subjects (Bachis et al., 2012, 2016b). Thus, HIV, through gp120, may promote synaptic pruning by a combination of increased p75NTR activation and a decreased trkB function. More experiments are needed to fully understand these mechanisms.

## Data Availability

All datasets generated for this study are included in the manuscript and/or the Supplementary Files.

## Ethics Statement

All studies were in accordance with the Guide for the Care and Use of Laboratory Animals as adopted and promulgated by the U.S. National Institutes of Health. The protocol was approved by the Georgetown University Animal Care and Use Committee.

## Author Contributions

IM designed the experiments and wrote the manuscript. AS designed and performed the behavioral studies, analyzed the data, and helped with the writing of the manuscript. GA and SS performed the molecular biology experiments and analyzed the data. VA designed, performed, analyzed the *in vitro* experiments, and helped with the writing of the manuscript. PF assisted with the interpretation of the behavioral data. All authors reviewed the results and approved the final version of the manuscript.

## Conflict of Interest Statement

The authors declare that the research was conducted in the absence of any commercial or financial relationships that could be construed as a potential conflict of interest.
