# Supplementary material for: Reversal of Cognitive Impairment in gp120 Transgenic Mice by the Removal of the p75 Neurotrophin Receptor
Source: Front Cell Neurosci. 2019 Aug 30;13:398. doi: 10.3389/fncel.2019.00398 (PMC6730486; doi:10.3389/fncel.2019.00398)
Supplement: Supplementary file 1 [file Data_Sheet_1.PDF]

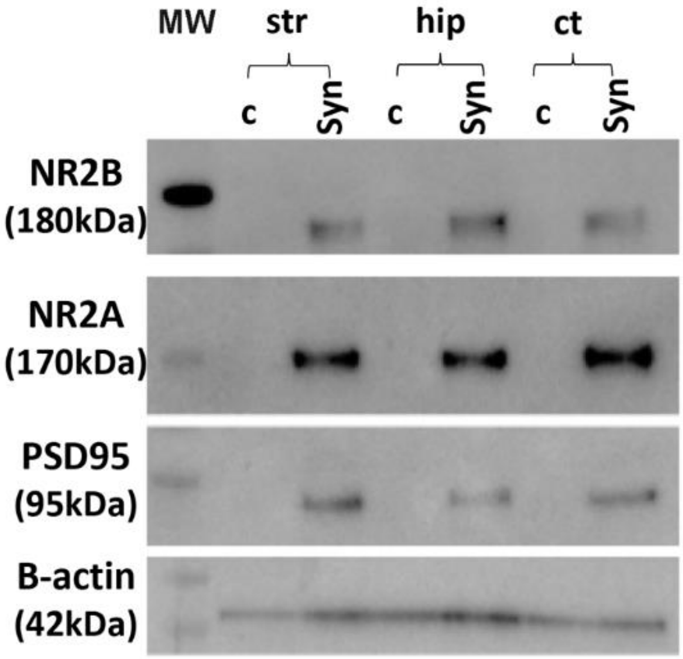

Figure S1. Representative western blot profile of synaptosome fractions of brain region homogenates. Cytoplasmic (c) and synaptosomal (Syn) fractions were prepared as described in Materials and Methods, from mouse striatum (str), hippocampus (hip) or cerebral cortex (ct). Lysates were loaded into a gel and analyzed using antibodies against NR2B, NR2A, and PSD95. Blot was stripped and reprobed with an antibody against beta-actin as a loading control. MW=molecular weight markers.
