# Supplementary material for: Reversal of Cognitive Impairment in gp120 Transgenic Mice by the Removal of the p75 Neurotrophin Receptor
Source: Front Cell Neurosci. 2019 Aug 30;13:398. doi: 10.3389/fncel.2019.00398 (PMC6730486; doi:10.3389/fncel.2019.00398)
Supplement: Supplementary file 2 [file Data_Sheet_2.PDF]

Supplementary Table 1: Morris Water Maze ANOVA results and p values for all pairwise comparisons within each training day

ANOVA for Acquisition Phase Training

Factor	*SS (Type III)*	*DF*	*MS*	*F (DFn, DFd)*	*p value*
Interaction	883.8	12	73.65	$F_{(12, 352)} = 0.8216$	p=0.6282
Training Day	34868	4	8717	$F_{(4, 352)} = 97.24$	p<0.0001
Genotype	3143	3	1048	$F_{(3, 352)} = 11.69$	p<0.0001
Residual	31555	352	89.65		

	Acquisition Phase Training Day				
	TD1	*TD2*	*TD3*	*TD4*	*TD5*
wt vs. gp120tg	0.9773	0.0015	0.0203	0.1045	0.1787
wt vs. $p75^{+/-}$gp120tg	0.2198	0.7127	0.9502	0.9993	0.9134
wt vs. $p75^{-/-}$gp120tg	0.9398	0.1799	0.7239	0.7500	0.8293
gp120tg vs $p75^{+/-}$gp120tg	0.0526	0.0222	0.0438	0.0811	0.4350
gp120tg vs $p75^{-/-}$gp120tg	0.9973	0.3694	0.2396	0.6010	0.6614
$p75^{+/-}$gp120tg vs $p75^{-/-}$gp120tg	0.0407	0.6894	0.9355	0.7698	0.9942

Note: Significant p values following Tukey's HSD are underlined.

ANOVA for Reversal Phase Training

Factor	*SS (Type III)*	*DF*	*MS*	*F (DFn, DFd)*	*p value*
Interaction	1720	12	143.3	$F_{(12, 338)} = 1.513$	p=0.1172
Training Day	16269	4	4067	$F_{(4, 338)} = 42.95$	p<0.0001
Genotype	3909	3	1303	$F_{(3, 338)} = 13.76$	p<0.0001
Residual	32006	338	94.69		

	Reversal Phase Training Day				
	TD7	*TD8*	*TD9*	*TD10*	*TD11*
wt vs. gp120tg	0.1372	<0.001	0.0300	0.5379	0.3910
wt vs. $p75^{+/-}$gp120tg	0.4635	0.7985	0.8912	0.8082	0.9791
wt vs. $p75^{-/-}$gp120tg	0.8191	0.7418	0.2763	0.6166	0.4957
gp120tg vs $p75^{+/-}$gp120tg	0.0005	<0.001	0.1006	0.9591	0.5644
gp120tg vs $p75^{-/-}$gp120tg	0.6172	<0.001	0.8298	0.9999	0.9999
$p75^{+/-}$gp120tg vs $p75^{-/-}$gp120tg	0.0787	0.9978	0.5978	0.9741	0.6745

Note: Significant p values following Tukey's HSD are underlined.
